# Art therapy focused on stimulating the emotional and expressive skills of the atypical children

**DOI:** 10.1192/j.eurpsy.2022.612

**Published:** 2022-09-01

**Authors:** L.-M. Hosu

**Affiliations:** CONSILIUL JUDEŢEAN CLUJ Direcţia Generală de Asistenţă Socială şi Protecţia Copilului, Centrul Comunitar JudeŢean Complex De Servicii Sociale Comunitare Pentru Copii și Adulţi Cluj, CLUJ-NAPOCA, Romania

**Keywords:** art therapy, social integration, raw art, outstanding skills

## Abstract

**Introduction:**

The role of the art therapist can be to identify the creative potential, to value it and to support social integration through art. Detecting and developing the outstanding and hidden abilities of the atypical child can lead to a normal behavior and to a better social integration.

**Objectives:**

Increasing self-esteem, through personal satisfaction, emotional development and the development of hidden and outstanding skills.

**Methods:**

Stimulating the child through the environment, works as a non-directive method during the art therapy session. Work environments offer various possibilities of expression, he chooses the materials to which he shows an interest, developing his own technique over time.

**Figure:**
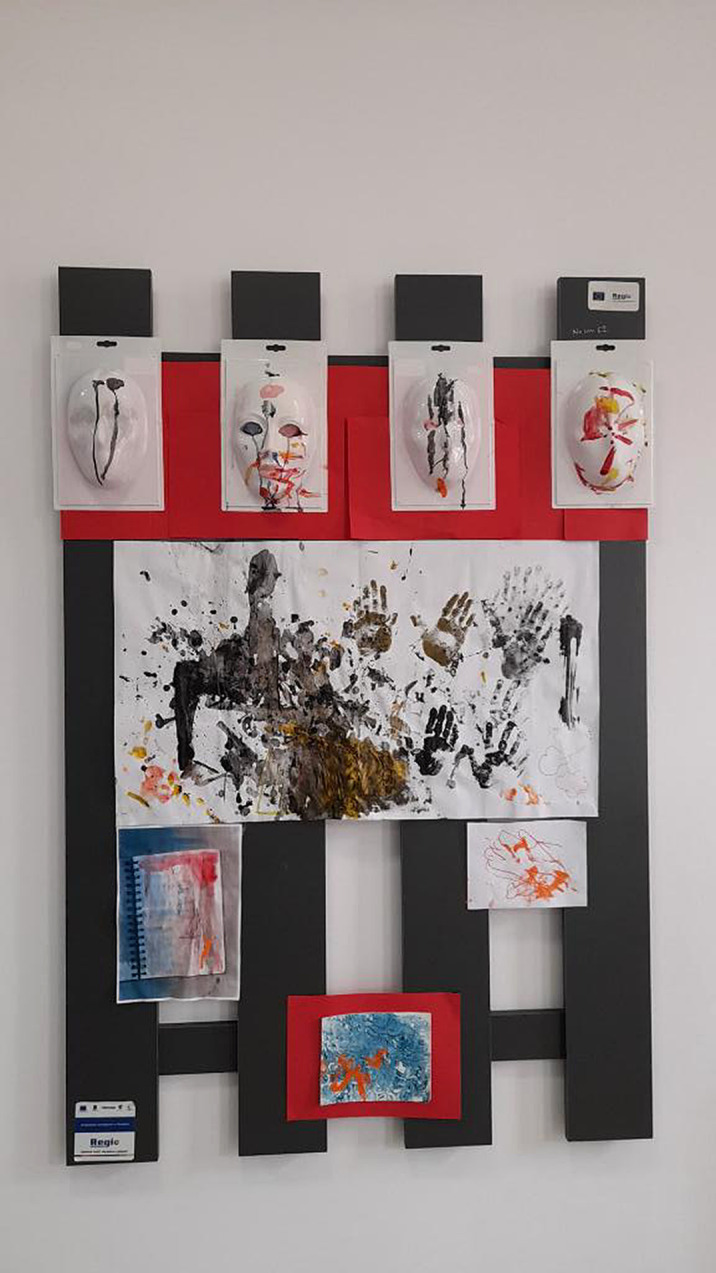

The child is encouraged during the art therapy sessions, by exhibiting the works and decorating the work environment.

**Figure:**
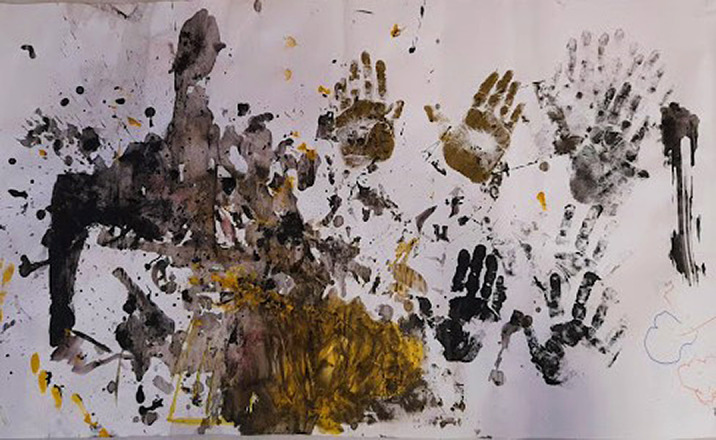

Through these non-directive methods, the evolution of visual thinking is accelerated. The chromatic diversification, the gestures in painting and the alternation of work techniques such as printing, graphic lines and dripping, are signs of a visual thinking. The child discovers the environment and interacts with it trough art.

**Figure:**
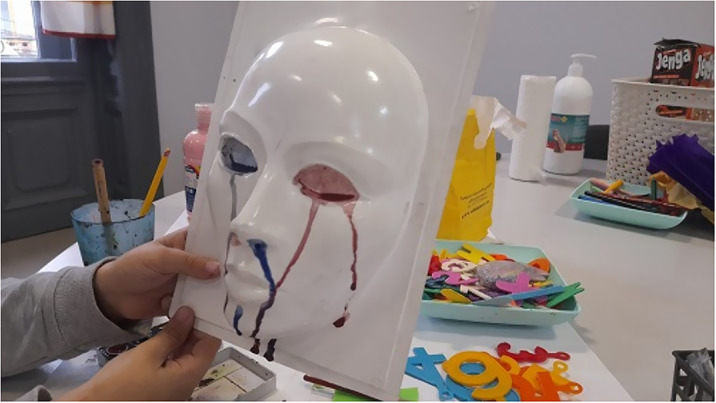

Observing the potential of the materials around him and also certains physical phenomena, such as a three-dimensional mask that allows the color to outline its volumes by draining it on the shape, the child uses consciously the properties of materials and the movement of the object.

**Results:**

Discovering artistic and decorative skills; Increased self-esteem; Interaction in the artistic environment and even verbal communication in cases of autism.

**Conclusions:**

Through art, the child can get closer to the social life.

**Disclosure:**

No significant relationships.

